# Severe asthma in South Africa: A literature review and management approach for primary care

**DOI:** 10.4102/safp.v63i1.5179

**Published:** 2021-01-29

**Authors:** Huibrecht C. Lion-Cachet, John M.M. Musonda, Olufemi B. Omole

**Affiliations:** 1 Department of Family Medicine and Primary Care, Faculty of Health Sciences, University of the Witwatersrand, Johannesburg, South Africa

**Keywords:** severe asthma, uncontrolled, difficult to treat, treatment-resistant, epidemiological context, primary care, management approach

## Abstract

Severe asthma in South Africa (SA) is underappreciated, especially in the primary care setting. This study highlights the epidemiological context of severe asthma as a phenotypic variant. Primary care practitioners, as first-contact medical providers, need grounding in the management of severe asthma based on the precision of diagnosis and negotiated along the 10-point strategy. The underdiagnosis and undertreatment of asthma ought to inform educational programmes and research in this country.

## Introduction

‘Wherefore is this *disease different* from *all other diseases*?’

*Maurice Pappworth* ^1^

Asthma still inflicts an underappreciated burden of disease on healthcare systems and the general society through the loss of workplace productivity and disruption in the functioning of families, especially with paediatric asthma. Over 330 million people have asthma globally.^2,3^ Since the year 2000, the prevalence of asthma plateaued in high-income countries (HICs), in contrast with upward trends observed in low-to-middle-income countries (LMICs).^2,3^ It was this ‘asthma epidemic’ that motivated epidemiological studies such as the International Study of Asthma and Allergies in Childhood (ISAAC) in 1995.^4^ The final phase of ISAAC, in 2002, indicated that in the international arena, the prevalence rates of asthma symptoms in the age groups of 6-7 years and 13-14 years were 11.5% and 14.1%, respectively.^4^ Similarly, the World Health Survey (WHS), conducted by To et al. between 2002 and 2003, estimated the prevalence of asthma symptoms amongst adults aged 18–45 years to be 14.4%.^5^

Meanwhile, the morbidity of asthma in HIC is being replicated in all South African populations.^6^ In Cape Town, ISAAC revealed that the 12 months prevalence of wheezing increased from 16% – 20% between 1995 and 2002. This increase could be suggestive of better awareness, screening and diagnostic techniques. The final phase of ISAAC was even extended to a rural population in Polokwane, where the 12 months prevalence of asthma symptoms was observed to be 18% in the adolescent group and 11.4% in the 6–7 year olds.^4,7^

To enable the comparison of prevalence rates of asthma between and within countries, it is necessary to use standardised measures in surveys conducted on a worldwide scale. Under the leadership of the United Nations, the Global Asthma Network (GAN) was established in 2012, which continued the worldwide asthma surveillance started by ISAAC. The GAN Phase I assesses prevalence, severity and risk factors in ISAAC participating centres and data will be available in 2020, updating the prevalence rates measured 15 years ago. Therefore, the prevalence rates referred to in this study are somewhat outdated, although of salutary standard.^8^ This article reviewed the literature on the epidemiology, phenotypes and management considerations of severe asthma and provided its evidence-based approach in primary care settings.

## What is severe asthma?

Asthma is regarded as a heterogeneous disease with various symptoms, such as a wheeze, shortness of breath, chest tightness and cough, which vary in intensity and over time.^9^ The earliest asthma guidelines were formulated with the perception that the diagnosis of asthma had to be followed by a grading of severity to guide treatment and prevent acute attacks. Whilst classification by degree of severity might give an impression of a fixed disorder, case definitions of asthma severity have varied over the past three decades. Consequently, guideline committees proposed that the current clinical control of asthma rather than asthma severity should be guiding treatment decisions.^3^

According to the National Education Prevention Programme, severity refers to the intensity of asthma, control of asthma is the degree to which symptoms abate with treatment and responsiveness of asthma is the ease of achieving a symptom-free result; hence, the terms ‘severity’ and ‘control’ incorporate both impairment (frequency and intensity of symptoms and functional deficiencies) and risks (possibility of future acute attacks, decline in lung function or lung growth in children and adverse effects of medication).^10^ Severe asthma, therefore, refers to the disease that is not controlled because of frequent or severe exacerbations, resistance to and control by treatment and its side effects, as well as the associated disease morbidity and mortality risks. The European Respiratory Society and American Thoracic Society (ERS/ATS) include the need for high-dose inhaled or oral corticosteroids as part of its definition of severe asthma in adults.^11^ Likewise, ‘severe’ asthma refers to the future risk of exacerbations, which has been even reported in patients with mild asthma unexpectedly succumbing to such an exacerbation of asthma.^12,13^

Severe asthma is usually subdivided into untreated severe asthma (uncontrolled), difficult-to-treat asthma and treatment-resistant severe asthma.^3,11^ Uncontrolled asthma indicates the presence of poor symptom control (frequent symptoms, reliever use, limitation of activity and night waking) and/or frequent or serious exacerbations a couple or more times annually requiring oral corticosteroids or hospitalisation, respectively.^14^ ‘Difficult-to-treat’ and severe ‘treatment-resistant’ asthma are categorised as ‘problematic-severe-asthma’.

## Severe asthma in South Africa: Challenges with screening and assessment

The ISAAC in Childhood developed user-friendly, written and video-assisted questionnaires to screen for the prevalence of asthma symptoms. Severe asthma, on the contrary, was screened for by eliciting a wheeze severe enough to limit speech, disturb sleep or initiate a visit to an emergency room. Although the tool developed by ISAAC to distinguish between asthma and severe asthma in children and adolescent participants was regarded as reliable, there is a lack of consensus on the definition of asthma and severe asthma. The different epidemiological surveys, such as the World Heath Survey and GAN Phase 1, confirm this discrepancy.^5,8^ Additionally, the formulation of defined primary outcomes in epidemiological studies has a substantial impact on projected prevalence rates. The conundrum in the various definitions of severe asthma complicates the interpretation of the results of meta-analyses. Inevitably, questionnaire-based studies may be open to participant interpretation compared with the investigator’s hypothesis.^15^

The Global Initiative for Asthma (GINA) Main Report 2020, published by the Global Strategy for Asthma Management and Prevention, acknowledges a limitation of clinical trials and epidemiological studies to formulate severity of asthma based on (prescribed) steps of treatment. Step 4–5 treatment is prescribed for patients with moderate-to-severe asthma. This dictum, however, is based on the presumption that the patient is actually receiving the treatment, and the more intense the treatment prescribed, the more severe the disease. Moreover, patients have to experience symptoms of uncontrolled asthma to meet the inclusion criteria for participation in epidemiological surveys,^14^ and patients have a different perception of the severity of their asthma compared to the doctors. The latter group, without prevail, categorises severe asthma according to the treatment prescribed, whilst patients view asthma severity by the frequency or intensity of the symptoms they experience.^16^

The ISAAC showed that the prevalence of severe asthma increased from 5.1% to 7.8% (odds ratio [OR] 1.58 (1.34–1.87) 95% confidence interval [CI], *p* < 0.001) amongst 13–14 year old participants in Cape Town, whilst in Polokwane the prevalence rates of severe asthma were noted to be 8% in the same age group; a follow-up epidemiological survey in Polokwane in 2009 indicated a 5.7% prevalence rate of severe asthma amongst a cohort of 6–7-year-old participants.^4^ There is a paucity of data, however, on severe asthma in middle-aged and older adults in South Africa because of the challenges with diagnosis (asthma - chronic obstructive pulmonary disease [COPD] overlap syndrome) and with conducting epidemiological surveys in this age group.^17^ One study, however, conducted by Green et al.^6^ in 2006 amongst the population living in the highly industrialised Durban metropole confirmed that children and adults both suffered from severe asthma with a prevalence rate of 16%.^6^

In 2008, a questionnaire-based survey was conducted by Green et al. amongst 710 patients in primary care across South Africa to assess morbidity of the disease in those on controller therapy. The findings revealed that characteristics of severe asthma, such as nocturnal wheezing up to four times a week (36.9%) and exercise-induced wheezing (56%), are observed in patients already on controller therapy. Although the questionnaire was not validated or translated into a wide range of languages, the data obtained were standardised at its source. This brought the dismal picture of poorly controlled and severe asthma to light. Although approximately 50% of participants reported persistence of severe asthma traits, they did not realise the severity of their disease.^18^ The deleterious effect of severe asthma on the quality of life was corroborated by various South African studies.^16,19,20^ For instance, a national lifestyle survey indicated that 64% of children and adults in South Africa reported absences from daily activities because of severe asthma, which is similar to rates in HIC.^18^

In LMICs, mortality rates of asthma have steadily been rising since 2000, resulting in 80% of the deaths in children worldwide. In 2014, Zar underlined the disjoint between the prevalence of asthma in South Africa (25th position globally), compared with our asthma mortality rates, which had the highest rate globally.^7,8^ Four years later, the GAN adjusted the figure of 280 deaths to about 100 deaths per 10^6^ of the population, the third highest spot overall.^8^ The case fatality rate for asthma was the fifth highest globally, with a projected rate of 18.5 per 100 000 asthmatics. South Africa ranked amongst the top countries in mortality figures, whether expressed as percentages of the total population or the asthma population.^8^ Reanalysis of ISAAC data by Green et al. revealed that whilst asthma was more prevalent in the affluent households, the burden of morbidity and mortality lies within impoverished households.^4^

## Severe asthma phenotypes in South Africa

According to Pavord et al., ‘asthma’ is an umbrella term for various asthma symptoms.^1^ Similar clinical manifestations or observable traits or ‘phenotypes’ with different underlying mechanisms are referred to as ‘asthma endotypes’. All of these subtypes are still associated with the term ‘asthma’ alike. A phenotype of severe asthma will typically present because of interactions between the maturing immune system, atopic sensitisation and infections in early life, all happening in a genetically susceptible individual. It is possible to differentiate phenotypes linked to severe asthma in primary care by taking an accurate history (Table 1). The importance of phenotypes is that diagnoses may be confirmed by biomarkers and very specific treatment aligned accordingly.^1^

**TABLE 1:** Phenotypes linked to severe asthma.^1,2^

Phenotype	Characteristics	Use of corticosteroids
Allergic asthma	A strong family history of allergies, eczema, allergic rhinitis and/or reacting to drugs. Sputum reveals eosinophils (Type 2 inflammation)†	Responds well
Non-allergic asthma	It is not associated with atopy. Sputum has eosinophils, neutrophils and inflammatory cells	Does not respond well
Adult-onset asthma	Often presents in females, after adolescence. This is non-allergic asthma. Occupational asthma is possibly another variant	Responds readily to high doses
Prominent asthma symptoms	Observed in patients with body mass indexes of > 30. Sputum has very few eosinophils. Children born to obese mothers have a higher prevalence of this phenotype	-

*Source*: Adapted from Global Initiative for Asthma (GINA). Global Strategy for Asthma Management and Prevention: (2019 update) [homepage on the Internet]. 2019 [cited 2019 June 2]. Available from: https://ginasthma.org

† , Type 2 comorbidities: nasal polyps and atopic dermatitis.

### Determinants of severe asthma

Factors that influence the risk of developing severe asthma phenotypes may originate from either the host or the environment (Table 2).

**TABLE 2:** Determinants of risk networks underlying severe asthma phenotypes, South Africa.

Host factors	Environmental factors
Genetic (multiple) Pathophysiology (endotypes) Increase in allergies Rhinitis and eczema *In-utero* In neonates, that is, Pre-term babies Low-birthweight babies Gender Obesity Age	Socio-economic characteristics in South Africa Stress Allergens: Indoor irritants (domestic mites, furs, irritants pollen and moulds) Occupational sensitisers and allergens (flour, laboratory rodents and paints) Infections (viral, bacterial and fungi) Microbiome Exposure to tobacco smoke (passive and active) Outdoor or indoor air pollution Diet

*sourcee:* Adapted from Global Initiative for Asthma (GINA). 2020 GINA Main Report. [homepage on the Internet]. 2020 [cited 2020 May 22]. Available from: https://ginasthma.org/gina-reports/

#### Host factors

Within phenotypic severe asthma, various asthma endotypes are present because of pathophysiological stages, such as inflammation, bronchial hyper-responsiveness and airway remodelling. If an individual has a strong family history of asthma, eczema and rhinitis, it could be pathognomonic of a severe phenotype of allergic asthma. Confirmatory sputum evaluation will reveal an abundance of eosinophils versus non-allergic endotypes, where a mixture of eosinophils and neutrophils in the sputum predicts resistance to corticosteroid treatment.^1,16^

#### Environmental determinants of severe asthma

The prevalence of respiratory allergies, in both urban and rural areas of South Africa, is increasing because of constant internal and external migration, resulting in rapidly changing lifestyles.^20^ As early as 1979, South Africa showed an urban-rural gradient in the distribution of asthma, whereas more recent surveys have indicated reductions in this gradient and that all populations and socio-economic groups are affected.^6^

A secondary analysis of the WHS in 2017 indicated an association between wheezing and social disconnection in Africa.^21^ The exact link between asthma, severe asthma and socio-economic deprivation remains complex. However, in an epidemiological survey involving 6000 poor urban and rural South African children, Yakubovich et al. showed that adverse socio-economic and psychosocial circumstances affect asthma prevalence and severity to a larger extent than did the urban-rural difference; poverty is linked to early-onset asthma and phenotypically severe asthma.^22^

Challenges in access to care, government policies, adverse early childhood development and inappropriate health-seeking behaviour contribute to the severity of asthma in South Africa.^21^ Consequently, asthma increases during weekends and whenever care may not be immediately accessible because of transport costs, long waiting times linked to staff shortages and associated losses in income.^23^

#### Modifiable risks

In a retrospective case-control study, 21 participants were admitted to the Red Cross War Memorial Children’s Hospital ICU in Cape Town. These patients experienced ‘seasonal’ acute asthma attacks. Their 65 admissions over 14 years amounted to 15.6% of all ICU asthma admissions, whilst the control group include patients with non-seasonal, severe asthma admissions. Although this was a small study, the seasonal asthma group formed a distinct subpopulation of severe asthma patients, some of whom had a family history of fatal asthma; they also had positive radio-allergosorbent tests to *Cladosporium* sp., *Aspergillus* sp. and grass pollen, significantly more often than the control group. Seasonality may prove to be a risk factor for asthma mortality.^6^

Rhino- and respiratory syncytial viruses are known triggers of wheezing in children. A specific gene expression enhances rhinovirus binding, precipitating asthma attacks. Impaired innate antiviral immunity with diminished interferon induction to rhinovirus has been reported in both children and adults with severe asthma. Upper respiratory tract infections often peak at the beginning of school terms, and so does asthma incidence.^1^ Pulmonary tuberculosis, bacterial, viral and atypical pneumonias confound the diagnosis of asthma in South Africa where human immunodeficiency virus (HIV) is endemic and overlaps with these infections.^24^ Finally, medications such as angiotensin inhibitors, b-stimulants also as eye drops, non-steroidal anti-inflammatory drugs and non-prescription aspirin are prescribed in asthma exacerbations, and therefore, in severe asthma.^14^

#### ‘Untreated, severe’ asthma in low-to-middle-income countries and South Africa

The World Health Organization (WHO) reserved a category for ‘untreated severe asthma’ in LMICs. This represents a distinct class of patients presenting with uncontrolled asthma who are not taking any ‘controllers’. In general, this is a situation created by the unavailability of asthma controllers, specifically inhaled corticosteroids (ICS) in low-resource settings.^4,8^ The GINA Main Reports since 2018 confirm the continued existence of this category, following pilot studies conducted by the International Union Against Tuberculosis and Lung Diseases (UNION) in several LMICs.^2,14,16^

Technical measures to be applied by healthcare workers were published in the UNION Asthma Guide, with the aim of minimising the severity of asthma and frequency of emergency room visits during exacerbations.^14^ As the underutilisation of ICS featured recurrently in the presentation of severe asthma, the UNION summoned an international collaboration with GINA and WHO in order to make controllers affordable in under-resourced regions; the services of the Asthma Drug Facility were contracted into this collaboration.^8^ Added to the UNION initiative, the GAN efforts ensured that health authorities and governments in LMICs stayed informed of gaps in the availability of ICS, now regarded as essential medication in the management of asthma.^8^

The combination of ICS and rapidly acting long-acting *β*-agonist (LABA) was added to the WHO’s Essential Medicines List (EML) for primary care facilities in 2018.^8^ Although South Africa has access to a well-developed public health system, and therefore to the EML, few clinicians in primary care prescribe ICS+LABA combination, perpetuating a lack of seamless access to these inhalers. Additionally, patients who have no access to the public sector have to privately purchase these costly inhalers.^25^

In LMICs, a comparison between the prevalence of asthma symptoms and doctor diagnosed asthma suggests that, at the population level, as many as 50% of cases may be undiagnosed.^14^ Jose et al. conducted a comprehensive systematic review of literature in the US National Library of Medicine up to 2012 and found the diagnostic accuracy of respiratory disease by general practitioners to be low, that is, asthma diagnosis varied from 54% underdiagnosis to 34% over-diagnosis. Despite the heterogenicity of the global articles reviewed in the meta-analysis, the study fulfilled its aim to illustrate the diagnostic challenge respiratory disease presents to primary caregivers.^26^

Mash et al. also reported that in the Western Cape, South Africa, missed diagnosis often resulted in untreated asthma. Overall, the promotion of regular peak expiratory flow measurements, written asthma action plans and inhaler technique reviews occurred seldomly during follow-up visits for chronic asthma patients at primary care clinics in the Western Cape.^17^

## A management strategy for severe asthma

As the unexpected evidence that short-acting bronchodilator (SABA) monotherapy was associated with increased mortality and asthma exacerbations, the strategy was to retrospectively alter the diagnosis of ‘mild’ asthma after the occurrence of a life-threatening event to that of phenotypical severe asthma.

The strategy reports by GINA in 2019 and 2020 represent a fundamental change in the approach to asthma in adults. Patients with mild asthma experience loss of asthma control more than twice a month, and therefore, the risk of severe exacerbations remains. Consequently, the recent global key recommendation by GINA was that these patients would need ICS whenever SABA’s are prescribed.^4^ Preference is given to combinations of ICS and LABA’s as needed, as they have proven to be as efficacious as twice daily ICS plus SABA’s as-needed to prevent asthma exacerbations.^16^ In children younger than 5 years, the approach, however, is to give a trial of controller therapy for disease presentation suggestive of severe asthma and after alternative diagnoses have been excluded. Long-acting β-agonist’s are not recommended in this age group.^14^

The recent GINA recommendation is based on the significant findings from the Symbicord Given as Needed in Mild Asthma (SYGMA) 1 and 2 trials. Symbicord Given as Needed in Mild Asthma 1 included an additional study group randomised to SABAs as-needed, thus randomly assigning participants to three treatment groups. Comparatively speaking, SYGMA 2 had two groups, that is, Symbicord as-needed and twice daily ICS plus SABA as-needed. In both multinational studies, carried out over 52 weeks, 3000 participants were randomised in a double-blind fashion, with the use of double-dummy inhaler. South Africa was represented by a 10% portion of the total participants. The primary aim of SYGMA 1 was to show longer term superiority of Symbicord therapy over SABA alone in the maintenance of mild asthma control whilst preventing exacerbations.^27^ Symbicord Given as Needed in Mild Asthma 2 was a pragmatic study, with the main aim of preventing exacerbations of asthma, and this succeeded to show equality in both study groups. In this regard however, regular ICS was superior in the control of mild asthma symptoms. More work is ongoing as the jury is still out as to whether the Symbicord as-needed regimen truly results in lower steroid exposure and improved real-world patient compliance.^28^

The Symbicord Given as Needed in Mild Asthma 1 and 2 prioritised the prevention of severe asthma mortality over control of mild asthma. This aim was also set by a another couple of high-quality trials.^29,30^ One of these was a systematic review and meta-analysis published in JAMA, 2018, comparing Single Maintenance and Reliever Therapy (SMART) in the form of the ICS+LABA combination used as a controller and reliever with ICS+LABA used as controller only in adult asthmatics. The former regimen resulted in an overall lower risk of asthma exacerbations.^31^ Indirect evidence from earlier trials also advocated the option of early use of the as-needed ICS+SABA’s combination to lower the future risk of mortality.^16,32,33^ In a commentary by the Lancet Commission on the GINA Report of 2018, the suggestion was that ideally the doses of corticosteroids in severe asthma should be biomarker driven to optimise control; no longer should it be ‘more corticosteroids in more lungs, but more inhaled steroids in the right lungs’.^1^

## Management strategy: Severe asthma phenotype in South Africa

Recommendations for the approach to severe asthma are formulated in the following section, based on the GINA Main Report 2020, as resource and intended for adaption in the local primary care settings. Insights gained from multicentre epidemiological studies and the South African guidelines were constructed into a treatment strategy.^14^

The eventual aim of the management of severe asthma is to achieve symptom control and to minimise the risk of future asthma-related mortality. Although guidelines for acute and chronic asthma in adults and children were published by the South African Childhood Asthma Working Group (SACAWAG) and the South African Thoracic Society, there are currently no allocated guidelines for severe asthma in South Africa.^34,35,36^ The methodology followed by experts to compile the guidelines was to systematically review the available high-quality scientific, international literature. Recommendations with regard to the management of asthma are graded as evidence levels A, B, C and D, with permission of the GINA. Furthermore, pre-existing interests were declared by the working groups, as pharmaceutical companies were involved in sponsoring some meetings during the development of these documents.^34^

### Category 1: Uncontrolled (untreated, undiagnosed) severe asthma

In the largest survey on chronic diseases across the globe, the WHS noted that amongst people with clinical asthma, one in two reported wheezing in the previous 12 months, but one in five had never received any asthma treatment.^21^ Commenting on the WHO’s consultation on severe asthma, Bush and Zar pointed out that untreated, severe asthma in LMICs is mainly caused by two factors: inaccessibility of ICS and spacers in under-resourced settings, and failure to diagnose asthma.^3^

#### Access to appropriate inhaled corticosteroids/inhaled corticosteroids combinations

A high-impact intervention in a low-resource setting is to ensure that affordable and low-risk medications are available as standard of care, embedded in local clinical practice. In terms of the high prevalence of severe asthma and mortality rates in South Africa, indicators for improving the quality of care need to be identified. Recommendations by practitioners should be addressed whilst balancing the care gaps and needs in the current care delivery. A working group is often best positioned to select and implement the main goals of such a quality improvement project and to develop strategies around barriers to change. Setting modest objectives will provide the initiative to enable the early recognition of severe asthma, aided by simple practice tools, and to improve seamless access by patients to appropriate medications. Often organisational problems because of lack of communication between caregivers and health practitioners need to be addressed in this regard. Implementation requires the involvement of professional groups and other stakeholders taking the socio-economic challenges and cultural conditions into consideration.^37^ Financial backing should be procured at the level of the National Department of Health, funding agencies, industry and professional societies.^14^

#### Considering asthma in the diagnosis of respiratory conditions in South Africa

In low-resource settings, respiratory symptoms, such as cough, shortness of breath and chest tightness, should follow a syndromic approach. The onset and duration of symptoms need to be assessed, and specific questions on fever and rigours, drenching night sweats, loss of weight, painful respiration and haemoptysis are added. The differential diagnosis of heterogenous respiratory symptoms includes chronic infections, such as tuberculosis associated with HIV/AIDS, as well as opportunistic fungal lung infections, which need to be ruled out before asthma and COPD are considered.^14^ For asthma, variable airflow limitation should be demonstrated, and hence, in low-resource settings, the WHO advocates documentation of the symptoms and response to a therapeutic trial with regular ICS and/or oral corticosteroids and SABA’s as-needed for 1 week. Measuring the peak expiratory flow before and afterwards will confirm the diagnosis of asthma. Simplifying this diagnostic process and management approach in primary care facilities will lead to the eventual recognition of severe asthma.^14^

### Category 2: Problematic severe asthma

#### Difficult-to-treat asthma subcategory

This is a category of patients with uncontrolled asthma despite medium- or high-dose ICS and other controllers; sometimes maintenance with oral corticosteroids or other treatment (GINA step 4 or 5) is needed to control asthma symptoms or prevent exacerbations.

#### Investigate

Before invasive investigations and maintenance, high-dose oral corticosteroid prescription is considered. The differential diagnosis of asthma and concomitant comorbidities should be addressed at every level of the staged approach to severe asthma, especially in the primary care setting.^35^

#### Is the diagnosis not asthma in the first place?

It is necessary to exclude conditions mimicking asthma in adults and children, taking into consideration that severe asthma is differently defined in preschool and school-aged children, each having associated differential diagnosis. Whilst preschool children often suffer from episodic viral wheezing, cystic fibrosis needs to be excluded in older children. In adults over the age of 40 years, COPD has to be distinguished from asthma, or it may be found to be coexisting as the COPD - asthma overlap syndrome.^38^

Clinical symptoms typical of asthma, such as dyspnoea, cough and wheeze, could undoubtably be because of an alternative diagnosis or comorbidity. A careful history and physical examination might reveal that dyspnoea is because of obesity or cardiac disease, cough part of an upper airway cough syndrome or angiotensin-converting enzyme (ACE)-inhibitors and wheeze embedded in COPD or bronchiectasis. Red flag symptoms are those suggestive of bronchiectasis, infective endocarditis and multiple comorbidities necessitating referral to a tertiary level of care.^14^

#### Is a comorbidity contributing to the symptoms?

Most commonly missed are allergic rhinosinusitis, obesity, obstructive sleep apnoea and gastro-oesophageal reflux disease. It must be noted that asymptomatic gastro-oesophageal reflux is unlikely to be the cause of uncontrolled asthma.^15,39^

Anxiety and depression are common comorbid conditions in difficult-to-treat asthma. Compounding socio-economic stressors in the South African environment are proven to affect adherence and risk for exacerbations.^22^ Utilising a psychologist or a multidisciplinary team is often needed.^14^

#### How can the diagnosis of asthma be confirmed?

The role of spirometry in difficult-to-treat asthma: The Adult Primary Care Guideline of 2019-2020 forms part of the Ideal Clinic Services Management. The APC tool underlines the necessity of spirometry at primary care level in order to distinguish COPD from asthma.^40^ The GINA Report (2020) similarly states that in low-resource settings, FEV1 and FVC should be recorded at least once during the course of treatment of patients with chronic airway disease because of the universal association between restrictive spirometry and poverty. Based on the South African literature, the Thoracic Society published a position statement on the management of COPD in 2019, and their third key point reiterates the essential role of spirometry in the early diagnosis of COPD.^41^

Whilst WHO regards peak expiratory flow as an essential tool in the Package of Essential Non-Communicable Diseases Interventions in low-resource settings to demonstrate variable airflow limitation in asthma, there is a definite role for spirometry in the assessment of severe asthma in primary care settings. The proposal made by the GINA Report, 2020, supports the availability of spirometry in primary care and building the capacity of healthcare workers in these settings.^14,42^ Although spirometry does not link accurately to asthma symptoms, it is of value to measure a decline in lung function in those with frequent asthma exacerbations, especially amongst children.^43^

#### Look for factors other than asthma, contributing to exacerbations

The prescribed medication has to be reviewed in asthma proving to be difficult to treat. Suboptimal adherence occurs in 75% of asthma patients. Asked about frequency of use:

[*F*]or most people it is difficult to use their inhaler as prescribed, how many days a week do you use yours?^16,44^ (p. 44)

The use of incorrect inhaler technique has been observed in approximately 80% of cases:

Ask: ‘*show me how you use your inhaler?’*^14^

Reviewing the metered-dose-inhaler technique of a patient at follow-up visits with the mnemonic: *Confirm choice, check and correct*:

- *Confirm choice* of an inhaler is acceptable to the patient; for instance, change to the ICS+LABA combination in patients with frequent attacks or who are having difficulty with concurrent use of multiple inhalers.^14^
- *Check* and *correct* inhaler technique by demonstrating the use of a spacer, or if unavailable a low-cost spacer (cool drink bottle spacer). Check understanding with a teach-back system.^14^

The fact medication side effects contribute to non-adherence calls for emphatic exploration of the challenges posed by inhaler use in the patient. Often the primary carer contributes to non-compliance by an attitude of indifference.^14^ An example is the overuse of SABAs promoting beta-receptor down-regulation lowering treatment response and, in turn, leading to higher demand. Dispensing 3 SABA cannisters per annum increases emergency department attendance, whilst prescription of an excess of 12 cannisters of SABAs annually (one per month) is in itself a red flag for an increased risk of suffering a fatal attack. So is the use of nebuliser machines. Reliever use as the main remedy, and neglecting controllers, should alert clinicians to airway remodelling and development of problematic severe asthma.^14^

It is important to review and manage *risk* factors in severe asthma that is uncontrolled, despite optimal pharmacological treatment:

Ask: *Are there modifiable environmental risk factors present*? Smoking and allergen exposure need to be kept in mind.^14^ Investigate the likelihood of other environmental exposures at home or work, such as moulds and noxious chemicals, and air pollution in or outdoors. Consider referral for allergen sensitisation checks, such as specific IgE or skin prick testing. Reassess the concomitant use of prescription and non-prescription medication, even eyedrops.^14^

Preventive measures, as in influenza and pneumococcal vaccines, might be necessary to lower the risk of exacerbations. A preventive approach is proposed for seasonal asthma and meteorological phenomena, such as thunderstorm asthma, whilst occupational asthma is a recognised severe asthma phenotype.^14^

#### Review and optimise management

Besides adjusting therapy to a reviewed diagnosis and optimising management of comorbidities and modifiable risk factors, Masekela et al. strongly advise primary carers to provide patients with an asthma self-management plan, preferably written out.^14,45^ SACAWAG convened in January 2017 to review and grade scientific literature on asthma self-management plans as an objective. The finding that thorough education benefits patients more than limited education added to the superiority of written asthma action plans over oral education, and graded as evidence level A. Patient motivation and adherence form a crucial aspect of asthma management, which is also indicated by a number of international guidelines. Culture-specific education for adults and children is more effective than generalised programmes in the cultivation of asthma knowledge, or improvement in the control of asthma and the quality of life, and prevention of exacerbations (evidence level B). Individualised asthma plans should be developed in accordance with the patient’s health-seeking behaviour.^45^

The GINA Patient Guide explains to the patient how to monitor danger symptoms, such as increased respiratory rate, decreasing oxygen saturation and/or a decrease in PEF; hence, patients are empowered to respond timeously to an exacerbation using an asthma self-management plan.^46,47^ Clinicians need to realise that education given during an emergency visit is not as effective as education during follow-up visits (evidence level A).^45^ After receiving emergency treatment for an asthma attack, patients need flagging for a definite follow-up date with the primary carer and were made aware that they are at risk of a fatal asthma attack in the future.^14^

Consider non-pharmacological add-on therapy, which includes physical exercise, a healthy diet and weight loss. Other strategies to follow are mucus clearance, breathing exercise and allergen avoidance, if applicable.^14^

Consider pharmacological add-on therapy, such as a trial of non-biologic agents or medium- to high-dose ICS, like tiotropium inhalers or leukotriene modifier, especially in children.^14^

The Asthma Guidelines Implementation Project (AGIP) adapted an asthma check to only five questions to assess response to treatment.

In the past few weeks, did you:

- Use your reliever three or more times a week? (except for one dose per day).
- Wheeze, cough or have a tight chest during the day, three or more times a week?
- Wake up because of wheezing, coughing or a tight chest in the night or early morning?
- Stop your usual activities because of asthma?
- Make an emergency visit to a healthcare worker? (even in the 2 months before?)

Three or more positive answers indicate uncontrolled asthma, and if all answers are negative, controlled asthma.^17^

Poor asthma control and high risk for future severe asthma attacks, according to the AGIP guidelines, are linked to the history of needing two or more courses of oral-steroid use for more than 3 days in the previous year, or serious exacerbations in need of hospitalisation or an ICU admission or ventilation in the previous year. Further tell-tale risk signals are the overuse of SABAs, psychiatric problems or previous anaphylaxis because of food allergy.^17^

#### Severe therapy-resistant subcategory

Therapy-resistant asthma is the second sub-category of persistent severe asthma (or sometimes as a subset of difficult-to-treat asthma).^48^ In this group, there is a persistence of severe asthma symptoms, despite adherence to optimised therapy and treatment of contributory factors, or worsening of asthma when high-dose treatment is stepped down. Two subsets are identified within the therapy-resistant category, namely, a refractory and a steroid-resistant subset, where the first group has uncontrolled asthma despite high-dose corticosteroids and the second group needs such high doses to control their condition that it results in adverse drug reactions, affecting the quality of life.^38^

In the Netherlands, an observational descriptive study was conducted to determine the comparative prevalence of difficult-to-treat and severe refractory asthma. Healthcare in the Netherlands is widely available and accessible because of the compulsory health insurance in the country. One finding was that severe refractory asthma had a lower than previously estimated prevalence of 3.6% or around 10 patients per 10 000 of the population. The study population was predominantly European ancestry and findings might not be applicable to a racially diverse population within a different healthcare system. The methodology was meticulously documented. A strength of the study was that a large number of participants were included as their medication use was registered in a countrywide pharmacy database. Inversely, because of lack of a consistent definition of severe asthma, the prevalence of such was difficult to obtain. The study was based on the international consensus statement published by the Innovative Medicine Initiative (IMI).^48,49^

The definition of severe asthma set by the ERS/ATS was different from the IMI definition, in that it included the patients in whom step down of high-dose corticosteroids led to resurgence of asthma symptoms. The expert committee tasked by ERS/ATS to compile a guideline on the management of severe asthma categorised the strength of recommendations using the Grading of Recommendation, Assessment, Development and Evaluation (GRADE) scale. Consequently, recommendations were categorised as recommended or suggested for discerning future use, increasing the applicability to primary care settings, even though the guideline was primarily intended for pulmonologists. Nevertheless, the literature search revealed the paucity of randomised control studies with low-risk bias. Therefore, a number of the clinical recommendations were based on indirect evidence from studies on participants with moderate-to-severe asthma.^11^

Individualised management of this small group of patients could lead to improved asthma control. The patients are assessed and treated for severe asthma phenotypes. Aspirin-precipitated asthma responds well to leukotriene modifiers. Where Type 2 inflammation resistant to low-dose ICS is suspected, the blood eosinophils may be assessed; this is to guide add-on therapy or lifestyle modification^14^ (see Table 1).

Low-dose macrolide therapy is regarded as specialist therapy for severe asthma with a predominance of neutrophils in the sputum. The extent to which asthma treatment can be individualised depends on the health system and the clinical context, cost and available resources.^14,38^ Patients with treatment-resistant severe asthma need referral to a specialist centre because primary care in South Africa is not equipped to measure biomarkers or dispense the newer and more expensive biological agents. Most research activity on individualised treatment is focused on severe asthma.^45^

Masekela et al. justifiably ask clinicians to ‘look beyond the magic bullet’ of new biologic therapies in problematic severe asthma, and rather ensure that patients have written-out asthma action plans. Although a personalised asthma action plan does nothing to improve lung function, it results in fewer days lost from school or work and less emergency department visits, even hospitalisations. The patients using it had less sleep disturbance because of asthma and less need for rescue medication (evidence level A).^45^

In summary, severe asthma management at primary care level should be negotiated along a 10-point plan:^16,35,44,47^

- No patient with asthma should use a SABA daily without an ICS.
- Regard ‘severe asthma’ as ‘severe untreated asthma’. To minimise risk, use mnemonic ‘ARM’:
  ■ *Assess*: inhaler technique, adherence to prescribed medication;
  ■ *Review*: diagnosis, comorbidity, irritants, sensitisers and/or psychosocial stressors;
  ■ *Monitor*: response to interventions.
- Assess the inhaler technique by the ‘*Confirm choice, Check and Correct*’ *-*mnemonic.^50^
- Assess compliance: Normalise the challenges of inhaler use, before asking for frequency of use by days per week.
- Introduce a spacer (or the ‘500 ml-cold-drink-bottle-spacer’ intervention) as a routine self-monitoring of asthma symptoms.
- The role of the peak flow meter is akin to that of a glucometer in diabetes mellitus.
- Clinicians and patients must recognise acute asthma flare-ups as a red flag for future asthma-mortality risk.
- Monitor severity and risks for flare-up using five question check by AGIP.
- A written asthma action plan for every patient relevant to his or her asthma severity is the gold standard.
- Referral to a tertiary level of care promotes access to endo-phenotyping and biological agents.

## Key take home messages

### Conclusion

As in other LMICs, South Africa is experiencing a silent epidemic of ‘untreated severe asthma’, rather than severe asthma, which is perfectly modifiable at primary healthcare level. Severe asthma in South Africa has been largely under-appreciated, even though it is the fifth highest mortality rate, globally, amongst young South Africans. The disease is characteristically found in pockets of affluence and poor-resourced areas along the historical racial segregation in South Africa. Doctors need to recognise an acute asthma attack as a red flag for fatality, and patients and their families must be made aware of this. The traditional 10-point asthma plan should be used by healthcare providers in primary care settings to optimise the care of individual patients and be reviewed at least annually.
